# Cost-Effectiveness of Apomorphine Sublingual Film as an “On-Demand” Treatment for “OFF” Episodes in Patients with Parkinson’s Disease

**DOI:** 10.36469/jheor.2021.29488

**Published:** 2021-11-17

**Authors:** Andrew Thach, Noam Kirson, Miriam L. Zichlin, Ibrahima Dieye, Eric Pappert, G. Rhys Williams

**Affiliations:** 1 Sunovion Pharmaceuticals Inc.; 2 Analysis Group, Inc.

**Keywords:** apomorphine sublingual film, apomorphine hydrochloride injection, cost-effectiveness analysis, levodopa inhalation powder, “off” episodes, parkinson’s disease, “on-demand” treatment

## Abstract

**Background:** “On-demand” treatments approved in the United States (US) for “OFF” episodes in Parkinson’s disease (PD) include apomorphine hydrochloride injection (SC-APO), apomorphine sublingual film (APL), and levodopa inhalation powder (CVT-301). APL received US approval in 2020, and its cost-effectiveness has not been compared with SC-APO and CVT-301.

**Objective:** To develop a cost-effectiveness analysis model comparing APL versus SC-APO and CVT-301 for treatment of patients with PD experiencing “OFF” episodes from a US payer perspective.

**Methods:** The model estimated total costs and effectiveness for each comparator arm, informed from the treatments’ pivotal studies or literature, over a 10-year horizon. Total and incremental patient costs (in 2020 US dollars), total time spent without “OFF” episode symptoms, and quality-adjusted life years (QALY) gained were summarized and compared. Incremental cost-effectiveness ratios for APL versus SC-APO and CVT-301 were estimated and expressed as incremental patient costs per patient QALY gained and incremental cost per “OFF” hour avoided. Scenario analyses varying inputs and including caregiver costs were also conducted.

**Results:** In the base case, APL had the lowest total “on-demand” treatment costs (42,095)comparedwithSC−APO(276,320; difference: –234,225)andCVT−301(69,577; difference: –$27,482) over the 10-year horizon. APL was also associated with the highest utility, with incremental QALYs of 0.019 versus SC-APO and 0.235 versus CVT-301. APL was dominant over CVT-301 in terms of incremental cost per “OFF” hour, and dominant over both CVT-301 and SC-APO in terms of incremental cost per QALY gained. In all scenario analyses, APL was dominant against both SC-APO and CVT-301, confirming the robustness of the base-case results.

**Discussion:** APL was dominant compared with both comparator arms, being less costly and more effective on average than SC-APO and CVT-301 in terms of QALYs. For SC-APO, cost-effectiveness of APL was driven by lower “on-demand” treatment costs and adverse event–related disutilities. For CVT-301, cost-effectiveness of APL was driven by lower “on-demand” treatment costs and substantially higher efficacy.

**Conclusions:** From a US payer perspective, APL represents a cost-effective option compared with SC-APO and CVT-301 for treatment of “OFF” episodes in patients with PD.

## BACKGROUND

Parkinson’s disease (PD) is a chronic, progressive neurologic disorder that results from the loss of dopaminergic neurons in the basal ganglia and subsequent disruption of thalamic connections to the motor cortex.^1^ PD is characterized by primary motor symptoms such as rest tremor, rigidity, bradykinesia, and postural instability^2^ as well as secondary (non-motor) symptoms, including mood and sleep disorders, cognitive changes, hallucinations and delusions, orthostatic hypotension, constipation, pain, and fatigue.^3,4^ The majority of PD cases arise sporadically,^5^ although there is evidence of genetic involvement in early-onset PD (ie, age at symptom onset is younger than 50 years).^6,7^ The worldwide prevalence of PD is approximately 0.3% in the general population aged over 40 years,^8^ and worldwide incidence estimates range from 8–18.6 per 100 000 person-years.^5^ As the incidence of PD increases with age,^9^ both incidence and prevalence of PD are projected to increase as the proportion of older people rises globally.^10,11^ In the US, approximately 1 million individuals are estimated to have PD. This number is projected to increase to 1.2 million individuals by 2030.^11^

The neuropsychiatric and motor symptoms of PD negatively affect patients’ health-related quality of life (HRQOL)^12,13^ and the life expectancy of patients with PD is shortened, particularly among patients with early-onset disease.^5^ There is currently no cure for PD, although a wide variety of pharmacologic and surgical treatments are available to manage the frequency and severity of symptoms.^14^ Dopamine replacement via levodopa-based treatment is the mainstay treatment; however, long-term use is frequently associated with motor fluctuations and dyskinesias.^15^

Motor fluctuations consist of periods when motor and non-motor symptoms of PD improve owing to a favorable response to a dose of carbidopa/levodopa (“ON”) and periods when the degree and duration of response is suboptimal and symptoms reappear or worsen (“OFF” episodes). Approximately 50% of patients with PD experience “OFF” episodes after 5 years of carbidopa/levodopa treatment, increasing to 70% beyond 9 years.^16,17^ Commonly reported motor symptoms during “OFF” episodes include tremor, slowness of movement, and balance problems, while frequently reported non-motor symptoms include anxiety, drenching sweats, slowness of thinking, fatigue, and akathisia.^18^ “OFF” episodes can significantly impact patients’ and caregivers’ HRQOL, employment, and work productivity.^13,19–21^ The onset of “OFF” episodes can be either predictable or sudden, adding to the complexity of treatment.^22^

Current pharmacological options for the management of “OFF” episodes include adjusting baseline carbidopa/levodopa treatment, adding “ON-extenders,” or adding “on-demand” treatments.^23^ “ON-extenders” are used as adjunctive daily maintenance treatments to extend “ON” time produced by doses of levodopa. These include dopamine agonists, monoamine oxidase-B or catechol-O-methyltransferase inhibitors, adenosine A_2A_ receptor antagonists, and extended-release amantadine. “On-demand” treatments are used when needed and can be administered during an “OFF” episode to rapidly reverse the episode and produce an “ON.” “On-demand” treatments currently approved by the US Food and Drug Administration (FDA) for the treatment of “OFF” episodes in patients with PD include apomorphine hydrochloride injection (APOKYN®; approved in 2004^24^), apomorphine sublingual film (KYNMOBI®; approved in 2020^25^), and levodopa inhalation powder (INBRIJA®; approved in 2018^26^). Apomorphine sublingual film and apomorphine hydrochloride injection require initial dose titration in a setting supervised by a health-care provider and with antiemetic premedication recommended 3 days prior to the first dose, while levodopa inhalation powder is administered at a single dose level without supervision.

The economic burden of PD in the United States is substantial, with combined direct and indirect cost estimated at nearly US$52 billion per year.^27^ Patients with PD and “OFF” episodes were found to have higher health-care resource utilization (HRU) and health-care costs than those without “OFF,” suggesting that “OFF” episodes contribute to the overall economic burden.^28^ Given the recent entry of apomorphine sublingual film to the treatment landscape for “OFF” episodes in PD, it is important to evaluate its economic value relative to the other two currently approved “on-demand” treatments. Thus, this study aimed to address the gap in the literature regarding the cost-effectiveness of the three currently approved “on-demand” treatments for “OFF” episodes in PD in the United States. To do so, we developed a cost-effectiveness analysis model of apomorphine sublingual film versus two other “on-demand” treatments (apomorphine hydrochloride injection and levodopa inhalation powder) for patients with PD experiencing “OFF” episodes.

## METHODS

### Model Overview

The cost-effectiveness analysis model was built from a pooled US Medicare and commercial payer perspective. It considered FDA-approved “on-demand” treatments used when needed to treat symptoms of an “OFF” episode in the target population, defined as adult patients with a diagnosis of PD treated with carbidopa/levodopa (with or without “ON-extenders”) and who experience “OFF” episodes. The “on-demand” treatments considered were apomorphine sublingual film, apomorphine hydrochloride injection, and levodopa inhalation powder. The model used a 10-year time horizon, which is consistent with a previously published cost-effectiveness analysis model in a similar target population with PD.^29^ It was developed using a microsimulation model with a 3-month cycle length, in order to best reflect the recurrent features of “OFF” episodes in patients with PD and the intended effect of “on-demand” treatments, and it was based on the apomorphine sublingual film pivotal study (CTH-300; ClinicalTrials.gov identifier: NCT02469090), which consisted of an open-label dose-titration phase and double-blind maintenance phase.^30^ Costs and benefits were discounted at a 3% annual rate over the time horizon.

The model was developed in R version 3.4.3 (R Core Team, Vienna, Austria, 2018). As this was a post hoc analysis of preexisting data, no institutional review board approval was required.

### Model Structure

**Figure 1.**
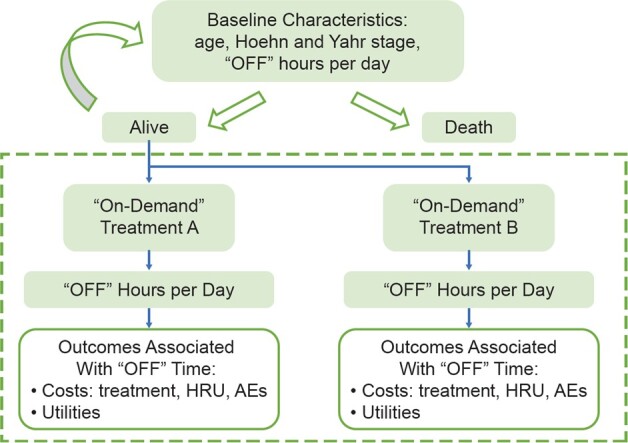
Cost-Effectiveness Analysis Model

The cost-effectiveness analysis model used a microsimulation structure to represent recurrent features of “OFF” episodes in patients with PD and the intended effect of “on-demand” treatments. This microsimulation model simulated the disease course of a cohort of 10 000 adult patients with PD receiving “on-demand” treatment for “OFF” episodes or no “on-demand” treatment over a 10-year time horizon. Figure 1 illustrates the model structure; patients entered the model with a randomly assigned baseline age, disease stage, and total number of “OFF” hours per day based on distributions informed by the CTH-300 study.^30^ Disease stage was described using the modified Hoehn and Yahr scale.^31^ Using these baseline characteristics, patients’ risk of death was estimated for each 3-month cycle. Probability of death was calculated from the US life tables^32^ with an adjustment based on the relative risk of death given the patients’ age and PD severity. Patients’ life status (alive or dead) was then simulated using the probability of death.

In each alive cycle, the patients’ total number of “OFF” hours per day was simulated based on distributions informed by clinical outcomes for each comparator arm. Patients were then assigned costs and utility values depending on their simulated “OFF” hours. “On-demand” treatment-related costs and adverse event (AE)–related costs/disutilities were applied in accordance with the comparator arm. The likelihood of treatment discontinuation was modeled based on a distribution of treatment duration informed by the CTH-300 study^30^ or literature.^33^ Patients who discontinued active “on-demand” treatment were assumed to follow the efficacy for patients on no “on-demand” treatment, which was assumed to have no “on-demand” treatment costs or AEs.

Baseline characteristics were updated after each 3-month cycle to inform the risk of death and other outcomes in the next cycle. Costs and utility values were aggregated over the 10-year time horizon for the simulated cohorts of patients receiving each type of “on-demand” treatment. The key cost-effectiveness result (incremental cost per quality-adjusted life year [QALY] gained) for apomorphine sublingual film versus each comparator arm was estimated. Table 1 includes a description of the key assumptions in the cost-effectiveness analysis model.

**Table 1.** Assumptions in the Cost-Effectiveness Analysis Model

**Aspect of Model**	**Key Assumptions**	**Justification**
Model states and transitions	Disease progression (eg, Hoehn and Yahr scale stage) and mortality are not affected by “on-demand” treatment	“On-demand” treatment is used when needed as an acute treatment of symptoms and is not expected to influence overall disease progression and mortality
	Probability of death was assumed to be based on age and disease stage at baseline and at the start of each model cycle	Presumably, patients that are older and/or have more severe PD (as per modified Hoehn and Yahr scale) have a higher probability of death
Costs	Costs associated with “OFF” time were independent of comparator arms, except indirectly through the effectiveness of “on-demand” treatment in reducing “OFF” time	It is not expected that costs associated with “OFF” time would be otherwise associated with treatment arms
	The underlying costs of PD were assumed to be the same across comparator arms and were not modeled explicitly	It is not expected that there would be systematic differences in the underlying costs of PD associated with different treatment arms
	The utilization and cost of maintenance (eg, carbidopa/levodopa) and “ON-extender” (ie, adjunctive) treatments (eg, dopamine agonists, COMT inhibitors, MAO-B inhibitors) were assumed to be the same across treatment comparator groups	The utilization of maintenance and “ON-extender” treatments may vary from patient to patient; however, it is not expected that there would be any systematic differences among the cohorts of patients initiating each of the respective treatments
	DACON assumed to be the same for all “on-demand” treatments	“On-demand” treatments are used when needed as an acute treatment of symptoms. Given that treatment costs are a function of how often patients use the treatment, the DACON is assumed equal to allow for more fair comparisons
Utilities	Utilities associated with “OFF” time are independent of comparator arms, except indirectly through the effectiveness of treatments in reducing “OFF” time	It is not expected that utilities associated with “OFF” time would be otherwise associated with treatment arms
	The underlying utility of PD was assumed to be the same across comparator arms and was not modeled explicitly	It is not expected that there would be differences in the underlying utility of PD associated with different treatment arms

Abbreviations: COMT, catechol-O-methyltransferase; DACON, daily average consumption; MAO-B, monoamine oxidase-B; PD, Parkinson’s disease.

### Model Inputs

Inputs for the cost-effectiveness analysis model are summarized in Table 2.

**Table 2.** Cost-Effectiveness Analysis Model Inputs

**Model Inputs**	**Category**	**Description**
Microsimulation inputs	Baseline characteristics	Age, Hoehn and Yahr stage, and “OFF” hours per day were informed by the pivotal trial of apomorphine sublingual film^30^ Mean (SD) age: 62.70 (8.95) years Mean (SD) “OFF” hours per day: 3.90 (1.81) Hoehn and Yahr stage probability Stage 1: 0.9% Stage 2: 73.2% Stage 3: 25.9% Stages 4 and 5: 0.0% Age and disease stage were updated at each 3-month cycle Probability of disease progression was derived from published literature^34^ Number of “OFF” hours was calculated as the product of patient-reported number of daily “OFF” episodes and typical duration of an “OFF” episode at patient’s baseline; values were updated at each 3-month cycle
	Efficacy	Changes in “OFF” time resulting from “on-demand” treatments: Apomorphine sublingual film: –1.32 hours^35^ Apomorphine hydrochloride injection: –1.32 hours^a^ Levodopa inhalation powder: –0.01 hours^35^
	Discontinuation	Three-month discontinuation rates were based on observed clinical trial data^30,33^ or computed for apomorphine hydrochloride injection as follows: s(t) = Xt +ty^b^ Apomorphine sublingual film: 27.8% Apomorphine hydrochloride injection: 15.8% Levodopa inhalation powder: 5.3%
	Mortality	Calculated from US life tables and adjusted for relative risk of death based on patient age and disease severity from published literature^36,37^
Cost inputs	Drug acquisition costs	WAC price per package^38^ Apomorphine sublingual film: US$787.50 Apomorphine hydrochloride injection: US $1100.00 Levodopa inhalation powder: US $997.50 DACON: 1.0 dose per day for all treatments No rebates or discounts assumed for any treatment No “on-demand” treatment was considered to have zero “on-demand” treatment costs
	Medical costs	Changes in per-patient medical costs were based on baseline HRU units, HRU associated with one extra “OFF” hour, and cost per unit of HRU (see **Online Supplementary Material Table S3** for values for hospitalization days, specialist visits, ER visits, and informal caregiver time^c^)^39,40^^,c,d^
	AE costs	AEs were assumed to occur in the first 3-month cycle, as patients who discontinue due to AEs would likely discontinue in the first cycle AE incidence rates were derived from published literature^30,41,42^ Unit cost for most AEs was based on HRU reported in the CADTH Pharmacoeconomic Report (see **Online Supplementary Material Table S2**)^42^ Total annual AE costs Apomorphine sublingual film: US$359.59 Apomorphine hydrochloride injection: US$110.73 Levodopa inhalation powder: US$510.35
Utility inputs	Utility associated with “OFF” time	Baseline utility values associated with “OFF” time were derived from published literature^43^/extrapolation methods “OFF” episode category I (1–4 hours): 0.643 “OFF” episode category II (4–8 hours): 0.555 “OFF” episode category III (8–12 hours): 0.467^e^ “OFF” episode category IV (12–16 hours): 0.379^e^ Nonlinear utility gain^f^ For the first “OFF” hour reduced: 0.044 For each additional hour after the first: 0.015
	Disutilities associated with AEs	HRQOL impact of specific AEs was incorporated as one-time disutilities during the first 3-month cycle Utility decrement values for AEs were obtained from published literature (see **Online Supplementary Material Table S2**)^39^ Total AE disutilities Apomorphine sublingual film: 0.014 Apomorphine hydrochloride injection: 0.001 Levodopa inhalation powder: 0.059

Abbreviations: AE, adverse event; CADTH, Canadian Agency for Drugs and Technologies in Health; DACON, daily average consumption; ER, emergency room; HRQOL, health-related quality of life; HRU, healthcare resource utilization; PD, Parkinson’s disease; SD, standard deviation; US, United States; WAC, wholesale acquisition cost.

^a^ As both “on-demand” treatments are different formulations of the same molecule (ie, apomorphine), both formulations were expected to have similar efficacy and thus, efficacy parity was assumed between apomorphine hydrochloride injection and apomorphine sublingual film.

^b^ Where t is week, s is share remaining on treatment at week t, X is the base rate for an exponential, and y is a linear “flattening” factor, assumed to be in parity with that computed based on apomorphine sublingual film data.

^c^ Based on data from the Adelphi Real World Disease Specific Programmes for PD.

^d^ Informal caregiver time was included in scenario 4 only and not the base model.

^e^ Values extrapolated based on utilities reported for “OFF” episode I and “OFF” episode II categories.

^f^ Utility per “OFF” hour was estimated based on the utilities reported for “OFF” episode I and “OFF” episode II categories, because baseline utility values for the other categories were extrapolated.

### Baseline Characteristics Inputs

Baseline data on age (mean [standard deviation]: 62.70 [8.95] years), “OFF” hours per day (mean [standard deviation]: 3.90 [1.81] hours), and Hoehn and Yahr stage probability (stage 1: 0.9%, stage 2: 73.2%, stage 3: 25.9%, stages 4 and 5: 0.0%) were based on the CTH-300 study (unpublished observations).^30^ Patients’ age and disease stage were updated at each 3-month cycle. The proportion of patients in Hoehn and Yahr stage 3 was calculated as the sum of patients in Hoehn and Yahr stages 2.5 and 3 at baseline. The probability of progression between Hoehn and Yahr stages was derived from the literature.^34^ Probability for each Hoehn and Yahr stage was calculated over the non-missing values for the Hoehn and Yahr stage reported for the overall modified intention-to-treat population (N=109). The number of daily “OFF” hours for each patient was calculated as the product of self-reported number of daily “OFF” episodes and the typical duration of an “OFF” episode at patients’ baseline. The number of “OFF” hours at each 3-month cycle was based on the efficacy of the respective “on-demand” treatments (see Efficacy Inputs below).

### Efficacy Inputs

The total amount of “OFF” time that a patient with PD experiences before and after on-demand treatment captures the extent of “OFF” episodes and the effectiveness and durability of “on-demand” treatments in relieving “OFF” symptoms. Data on “OFF” hour decrements owing to “on-demand” treatment were derived from a matching-adjusted indirect comparison of the efficacy of apomorphine sublingual film versus levodopa inhalation powder for the treatment of “OFF” episodes in patients with PD.^35^ The post-weighting, anchor-based mean changes in daily “OFF” hours from that matching-adjusted indirect comparison were –1.32 “OFF” hours with apomorphine sublingual film and –0.01 “OFF” hours with levodopa inhalation powder.^35^ The value for reduction in total daily “OFF” time for levodopa inhalation powder was derived directly from the levodopa inhalation powder pivotal study.^41^ Changes in daily “OFF” hours for apomorphine sublingual film were calculated from the CTH-300 study^30^ by multiplying the mean number of apomorphine sublingual film treatments per day (derived from patients’ home dosing and response diaries) and the imputed duration of “ON” per treatment episode (in-clinic data on patients’ “ON”/“OFF” status at discrete points in time).

Because of limitations in the pivotal apomorphine hydrochloride injection study,^44^ a similar anchor-based comparison could not be performed for apomorphine sublingual film versus apomorphine hydrochloride injection. In addition, both “on-demand” treatments are different formulations of the same molecule (ie, apomorphine). For these reasons, efficacy parity was assumed between apomorphine hydrochloride injection and apomorphine sublingual film (ie, a change in –1.32 “OFF” hours for apomorphine hydrochloride injection) for the model.

### Treatment Discontinuation Inputs

The apomorphine sublingual film 3-month discontinuation rate was calculated based on reported discontinuations due to treatment-emergent AEs.^30^ In the same pivotal study, patients discontinued treatment due to AEs until approximately week 12, after which the discontinuation rate remained mostly constant. Thus, the model assumed that patients did not discontinue the treatment after 12 weeks, equivalent to assuming that patients only discontinue in the first cycle. The apomorphine hydrochloride injection 3-month discontinuation rate was not available in the literature and was, therefore, computed assuming the survival on treatment function was given by: s(t) = Xt + ty, where t is week, s is the share remaining on treatment at week t, X is the base rate for an exponential, and y is a linear “flattening” factor. The flattening factor (y) was computed based on the apomorphine sublingual film data and was assumed to be the same for the comparator arms. The base rate, X, was calculated from the 1-month discontinuation rate for apomorphine hydrochloride injection and y. The levodopa inhalation powder 3-month discontinuation rate was based on LeWitt et al.^33,41^ The estimated 3-month discontinuation rates incorporated in the model were 27.8% for apomorphine sublingual film, 15.8% for apomorphine hydrochloride injection, and 5.3% for levodopa inhalation powder.

### Mortality Inputs

Probability of death was calculated from the US life tables^32^ with an adjustment based on the relative risk of death given the patients’ age and PD severity, informed by Liou et al.^36^ Mortality hazard ratios based on age and Hoehn and Yahr stage are shown in the Online Supplementary Material, Table S1.

### Cost Inputs

Costs considered in the model included “on-demand” treatment costs, “OFF” episode–related medical costs, and AE-related costs. Patients were assigned “on-demand” treatment costs based on their simulated overall survival and time on treatment using the treatment cost inputs outlined below. Medical costs were based on patients’ simulated overall survival, time on treatment, and number of “OFF” hours per day. A one-off AE-related cost was applied in the patients’ first 3-month cycle (by aggregating the data listed in the Online Supplementary Material, Table S2). Raw costs not reported in 2020 US dollars (USD) were inflated to 2020 USD based on the medical component of the Consumer Price Index.^45^

### Drug Acquisition Costs

For each treatment, the per-patient cost of treatment was based on the following components: average dose (for apomorphine hydrochloride injection), wholesale acquisition cost (WAC) price per package, total package size, and daily average consumption. The model assumed no rebates or discounts for any treatment. For apomorphine hydrochloride injection, the average dose (4.0 mg) was based on the simple average dose of studies included in the Canadian Agency for Drugs and Technologies in Health (CADTH) Common Drug Review, Clinical Review Report on apomorphine hydrochloride injection.^42,44,46^ For apomorphine hydrochloride injection and levodopa inhalation powder, the WAC price per package (US$1100.00 and US$997.50, respectively) and total package size (30 mg and 2520 mg, respectively) were derived from the IBM Micromedex RED BOOK.^38^ Daily average consumption (1.0 dose per day for all treatments) was based on internal assumptions. The WAC package price for apomorphine sublingual film (US$787.50) was based on information from the IBM Micromedex RED BOOK.^38^ Apomorphine sublingual film dosage does not affect the “on-demand” treatment costs because it is available at a flat package price regardless of dose per strip. No “on-demand” treatment was assumed to have zero “on-demand” treatment costs. “On-demand” treatment costs were applied over the simulated duration of treatment, accounting for treatment discontinuation and patient mortality risk.

### Medical Costs

While “on-demand” treatment costs account for the majority of the costs included in the cost-effectiveness analysis model, changes in medical costs owing to reductions in “OFF” time were also considered. Changes in per-patient medical costs were based on the following components: baseline units of HRU (ie, hospitalization days, specialist visits, emergency room visits, and informal caregiver hours), HRU associated with one extra “OFF” hour, and cost per unit of HRU. Baseline HRU and HRU associated with one extra “OFF” hour were calculated based on data from Adelphi Real World’s Disease Specific Programmes for PD. Cost per unit for medical resources was based on Palmer et al.^39^ Medical cost inputs are summarized in the Online Supplementary Material, Table S3. Units of HRU and changes associated with one extra “OFF” hour were normalized to 3-month periods used in the microsimulation model.

### AE Costs

Costs related to the management of key treatment-emergent AEs were considered in the model. AEs were assumed to occur in the first 3-month cycle to approximate the time in the titration period for apomorphine sublingual film and apomorphine hydrochloride injection (levodopa inhalation powder does not require dose titration). Patients were assumed to enter the model after they had been titrated to the optimal dose. AE incidence rates, listed in the Online Supplementary Material, Table S2, were extracted from pivotal study data for apomorphine sublingual film,^30^ from a CADTH Pharmacoeconomic Report for apomorphine hydrochloride injection,^42^ and from LeWitt et al for levodopa inhalation powder.^33^ All types of AEs related to swelling were grouped under “edema” for consistency with the levodopa inhalation powder and apomorphine hydrochloride injection pivotal studies.^30,44^ Because of the potentially differing definitions of dyskinesia across studies, this AE was excluded in order to be conservative. Unit costs for AEs, listed in the Online Supplementary Material, Table S2, were primarily based on HRU reported in the CADTH Pharmacoeconomic Report.^42^ For AEs not reported there, the model assumed AE-related HRU comparable to that reported in the CADTH Pharmacoeconomic Report. For apomorphine sublingual film, apomorphine hydrochloride injection, and levodopa inhalation powder, the total annual AE costs were estimated at US$359.59, US$510.35, and US$110.73, respectively.

### Utility and Disutility Inputs

Utility values were derived from a cost-effectiveness study in patients with PD by Lowin et al.^43^ EuroQol – 5 Dimensions data were reported by Hoehn and Yahr status and time spent in “OFF” episodes, and utility weights were estimated for each health state. Four categories of “OFF” hour benefit were constructed (“OFF” episode category I: 1–4 hours, “OFF” episode category II: 4–8 hours, “OFF” episode category III: 8–12 hours, and “OFF” episode category IV: 12–16 hours), per Lowin et al.^43^ Utility per “OFF” hour was estimated based on the utilities reported for “OFF” episode category I (utility: 0.643) and “OFF” episode category II (utility: 0.555), since utility values for other categories were based mainly on extrapolation rather than observed data. In the base case, the model assumed that half of the change in utility between “OFF” episode category I and “OFF” episode category II was attributed to the first “OFF” hour reduced, reflecting patients’ use of “on-demand” treatments when most needed. This assumption was modified in the scenario analyses described below. The nonlinear utility gain for the first “OFF” hour reduced was assumed at 0.044, and for each additional hour reduced, the utility was assumed at 0.015.

The HRQOL impact of specific treatment-emergent AEs were incorporated as a one-time disutility during the titration period (first 3-month cycle), following other published cost-effectiveness evaluations.^42^ Utility decrement values for AEs were obtained from Palmer et al.^39^ and consisted of 0.16 for dizziness and 0.18 for chest pain/pressure/angina. Subsequent effects of treatment-emergent AEs were captured largely through treatment discontinuation rates. Apomorphine sublingual film, apomorphine hydrochloride injection, and levodopa inhalation powder total AE disutilities were 0.014, 0.059, and 0.001, respectively.

### Model Outputs

#### Base Case

The model estimated the total costs and effectiveness for each comparator arm included in the model. Total and incremental patient costs and QALYs were summarized for each arm and for apomorphine sublingual film versus each comparator (apomorphine hydrochloride injection and levodopa inhalation powder). A detailed breakdown of costs by category were also reported (eg, “on-demand” treatment costs, treatment-emergent AE costs, medical costs). Health effects in the model were expressed in terms of QALYs gained and total time spent without “OFF” episode symptoms. Incremental cost-effectiveness ratios for apomorphine sublingual film versus apomorphine hydrochloride injection and levodopa inhalation powder were estimated and expressed as the incremental patient costs per-patient QALY gained and incremental cost per “OFF” hour avoided.

#### Scenario Analysis

As the microsimulation model captured the potential variation in the inputs and the resulting outputs, additional probabilistic sensitivity analyses, which are often performed for Markov cohort models, would not contribute meaningful insights. The following set of four scenario analyses were compared with the base case: (1) using efficacy inputs from a different indirect treatment comparison of apomorphine sublingual film and levodopa inhalation powder (unpublished observations); (2) assuming linear change in utility per “OFF” hour reduced (ie, each “OFF” hour reduced as a result of an “on-demand” treatment will cause the same gain in utility, as opposed to the baseline assumption of greater gain from the first hour of “OFF” episode reduction); (3) limiting the model horizon to 5 years; and (4) considering costs owing to caregiver burden in the model. The scenario analysis for caregiver burden considered informal caregiver time (hours spent caregiving) and associated costs (Online Supplementary Material, Table S3). The baseline number of caregiver hours (444.22 hours over the 3-month cycle) was derived from the overall mean caregiver hours reported in Findley et al.^29^ The incremental unit was calculated by taking the difference in mean hours for patients in “OFF” episode category IV and category I. Assuming this difference was linear with regard to “OFF” hours, it was divided by the “OFF” hours spread between the two categories (12 hours) to obtain the units associated with one extra “OFF” hour (18.8 hours). Cost per unit for caregiver hours was listed with the US Bureau of Labor Statistics.^40^

## RESULTS

### Base Case

The results of the base case analysis (using an annual discount rate of 3% for costs and utilities) are listed in Table 3. Over a 10-year time horizon, “on-demand” treatment costs were estimated at US$42 095 for apomorphine sublingual film, US$276 320 for apomorphine hydrochloride injection, and US$69 577 for levodopa inhalation powder (2020 USD). The differences in “on-demand” treatment costs were US–$234 225 between apomorphine sublingual film versus apomorphine hydrochloride injection, and US–$27 482 between apomorphine sublingual film versus levodopa inhalation powder. Because of higher discontinuation rates, patients in the apomorphine sublingual film arm incurred slightly higher medical costs compared with apomorphine hydrochloride injection (US$3403 vs US$3363; difference: US$40). Conversely, because of substantially higher efficacy, patients in the apomorphine sublingual film arm incurred lower medical costs relative to levodopa inhalation powder (US$3403 vs US$3627; difference: US–$224). The costs related to treatment-emergent AEs were US$367 (apomorphine sublingual film), US$530 (apomorphine hydrochloride injection), and US$114 (levodopa inhalation powder), with differences of US–$163 for apomorphine sublingual film versus apomorphine hydrochloride injection and US$253 for apomorphine sublingual film versus levodopa inhalation powder. Thus, apomorphine sublingual film had the lowest total “on-demand” treatment costs (US$45 865) compared with apomorphine hydrochloride injection (US$280 213; difference: US–$234 349) and levodopa inhalation powder (US$73 317; difference: US–$27 453) over the 10-year time horizon.

**Table 3.** Base-Case Results of the Model

	**Apomorphine Sublingual Film**	**Apomorphine Hydrochloride Injection**	**Levodopa Inhalation Powder**	**Apomorphine Sublingual Film vs Apomorphine Hydrochloride Injection**	**Apomorphine Sublingual Film vs Levodopa Inhalation Powder**
**Costs (2020 USD)**					
“On-demand” treatment costs (rebated)	$42 095	$276 320	$69 577	–$234 225	–$27 482
Medical costs	$3403	$3363	$3627	$40	–$224
Adverse event costs	$367	$530	$114	–$163	$253
Total costs	$45 865	$280 213	$73 317	–$234 349	–$27 453
**Effectiveness**					
Number of “OFF” hours	7732	7314	10 076	419	–2344
Life years	6.020	6.020	6.020	0.000	0.000
QALY, years	4.107	4.088	3.872	0.019	0.235
**ICER (2020 USD)**					
Incremental cost per “OFF” hour avoided	–	–	–	$559^a^	Dominant
Incremental cost per QALY gained	–	–	–	Dominant	Dominant

Abbreviations: ICER, incremental cost-effectiveness ratio; QALY, quality-adjusted life year; USD, United States dollars.

^a^ Costs and efficacy for apomorphine sublingual film are lower than those of the comparator.

Over the same time period, apomorphine sublingual film was also associated with the highest utility, with incremental QALYs of 0.019 compared with apomorphine hydrochloride injection (4.107 vs 4.088 QALYs, respectively) and 0.235 compared with levodopa inhalation powder (4.107 vs 3.872 QALYs) (Table 3). The number of “OFF” hours was highest for levodopa inhalation powder (10 076 hours) compared with apomorphine sublingual film and apomorphine hydrochloride injection (7732 and 7314 hours, respectively). All three treatments were associated with 6.02 life years. The incremental cost per “OFF” hour avoided was US$559 for apomorphine sublingual film versus apomorphine hydrochloride injection. Apomorphine sublingual film was dominant over levodopa inhalation powder in terms of incremental cost per “OFF” hour, and dominant over both levodopa inhalation powder and apomorphine hydrochloride injection in terms of incremental cost per QALY gained. Apomorphine sublingual film was projected to be dominant in 70% of patients versus apomorphine hydrochloride injection and 71% versus levodopa inhalation powder.

### Scenario Analysis

The results of scenario analyses are summarized in Table 4. Apomorphine sublingual film was dominant against both apomorphine hydrochloride injection and levodopa inhalation powder in all scenarios, including the scenario that incorporated caregiver cost burden (scenario 4). In that scenario, the incremental costs of apomorphine sublingual film versus apomorphine hydrochloride injection were US–$231 110 and those of apomorphine sublingual film versus levodopa inhalation powder were US–$45 719; the incremental QALYs gained were 0.019 and 0.235, respectively.

**Table 4.** Scenario Results of the Model

	Apomorphine Sublingual Film	Apomorphine Hydrochloride Injection	Levodopa Inhalation Powder	Apomorphine Sublingual Film vs. Apomorphine Hydrochloride Injection	Apomorphine Sublingual Film vs. Levodopa Inhalation Powder
Scenario 1: Efficacy data from an indirect treatment comparison of apomorphine sublingual film and levodopa inhalation powder^a^					
Total costs	$45 802	$280 139	$73 139	–$234 337	–$27 337
QALY, years	4.141	4.116	4.071	0.025	0.070
Incremental cost per QALY gained	–	–	–	Dominant	Dominant
Scenario 2: Assuming linear change in utility per “OFF” hour reduced					
Total costs	$45 864	$280 212	$73 317	–$234 349	–$27 454
QALY, years	3.983	3.961	3.871	0.022	0.112
Incremental cost per QALY gained	–	–	–	Dominant	Dominant
Scenario 3: Limiting the model horizon to 5 years					
Total costs	$30 918	$187 248	$48 874	–$156 330	–$17 956
QALY, years	2.731	2.702	2.578	0.029	0.153
Incremental cost per QALY gained	–	–	–	Dominant	Dominant
Scenario 4: Considering costs owing to caregiver burden					
Total costs	$484 956	$716 066	$530 675	–$231 110	–$45 719
QALY, years	4.107	4.088	3.872	0.019	0.235
Incremental cost per QALY gained	–	–	–	Dominant	Dominant

All costs are presented in 2020 USD.

Abbreviations: LY, life year; QALY, quality-adjusted life year; USD, United States dollars.

^a^ Scenario 1 used “OFF” hour decrements of 1.7 for apomorphine sublingual film and 0.8 for levodopa inhalation powder and assumed parity between apomorphine sublingual film and apomorphine hydrochloride injection (unpublished observations).

## DISCUSSION

Patients with PD often experience “OFF” episodes that can substantially impact HRQOL, ability to work, and caregiver burden.^19–21^ “On-demand” treatments provide benefits of rapid onset for relief of motor symptoms during an “OFF” episode and can be administered concomitant to or between doses of carbidopa/levodopa when needed. With the recent FDA approval of apomorphine sublingual film as an “on-demand” treatment for patients with PD who experience “OFF” episodes, it is important to assess its cost-effectiveness compared with the other two available treatments (apomorphine hydrochloride injection and levodopa inhalation powder) to help inform decisions by payers, patients, caregivers, and health-care providers. This study constructed a microsimulation cost-effectiveness analysis model from a US payer perspective with a 10-year time horizon, incorporating efficacy and cost inputs informed by data from the pivotal studies of the “on-demand” treatments or the literature. The results of the model indicated that apomorphine sublingual film was the dominant treatment choice, in terms of being both cost saving and resulting in improved health outcomes, relative to apomorphine hydrochloride injection or levodopa inhalation powder in the treatment of “OFF” episodes in patients with PD.

Considering both cost and utility outcomes, apomorphine sublingual film was dominant compared with both comparator arms, being less costly and more effective on average than apomorphine hydrochloride injection and levodopa inhalation powder. The favorable result of apomorphine sublingual film versus apomorphine hydrochloride injection was primarily driven by the large difference in “on-demand” treatment costs, which was coupled with a modest advantage in QALYs gained. While apomorphine sublingual film and apomorphine hydrochloride injection were assumed to be associated with equal reductions in daily “OFF” hours, the advantage in QALYs was the result of a more favorable AE profile (ie, lower AE-related disutility), which in turn offsets the higher rates of discontinuation with apomorphine sublingual film. The favorable result of apomorphine sublingual film versus levodopa inhalation powder was driven by higher efficacy of apomorphine sublingual film (as demonstrated by a larger reduction in daily “OFF” time from a prior indirect treatment comparison)^35^ and lower costs of treatment. These results were robust across four different scenario analyses that varied the inputs, limited the model horizon, or considered indirect costs owing to caregiver burden. The microsimulation findings indicated that apomorphine sublingual film was dominant across approximately 70% of runs against either comparator, and the various scenario analyses had little effect on the comparative findings.

As this is the first study exploring the cost-effectiveness of apomorphine sublingual film relative to apomorphine hydrochloride injection and levodopa inhalation powder, there are no other studies to directly compare with the present results. However, several cost-effective analyses have been conducted of PD treatments,^34,47–50^ as well as for treatments of “OFF” episodes, although the latter are primarily in Europe. A cost-effectiveness analysis by Walter et al reported that continuous subcutaneous apomorphine hydrochloride injection for patients with PD and “OFF” episodes was cost-effective versus other treatments available in the United Kingdom and Germany.^51^ A cost-effectiveness analysis by Lowin et al reported that carbidopa/levodopa intestinal gel was cost-effective to reduce “OFF” time versus standard of care in the United Kingdom based on QALY gained and the incremental cost-effectiveness ratio.^43^ Another cost-effectiveness analysis by Kalabina et al reported that carbidopa/levodopa intestinal gel was cost-effective versus standard of care in Scotland and Wales to reduce “OFF” time in patients ineligible for deep-brain stimulation or apomorphine hydrochloride injection.^52^ While these cost-effectiveness analyses used a Markov model as the basis for their analyses, the current study relied on a microsimulation approach that facilitates heterogeneity of individual patient outcomes. In addition, the microsimulation model does not assume any changes in disease progression owing to “on-demand” treatment. However, the other published models, while not focused on “on-demand” treatments, also incorporate “OFF” hours as well as common AEs and derived model inputs from pivotal studies and the literature.

The progression of PD differs in each patient, thus the cost-effectiveness of certain treatments and how they impact HRQOL or meet individual patients’ needs should be considered, with the goal of maximizing the time spent in less severe disease states. In addition, studies that consider other indirect costs, such as patients’ work loss or impacts on income, may be considered as avenues for future research.

A strength of this study is that the microsimulation approach provided opportunities to capture heterogeneity among individuals, especially regarding daily “OFF” time and disease stage.

### Limitations

This study is subject to several limitations, some of which are inherent to cost-effectiveness analyses. First, this model did not account for potential modifications to maintenance treatments for PD as a result of “on-demand” treatment utilization. Second, the cost inputs for “on-demand” treatment may not have taken into account all aspects of real-world treatment use. For example, wastage owing to priming with apomorphine hydrochloride injection could further increase the cost of apomorphine hydrochloride injection. Antiemetic pretreatment or treatment with antiemetics after dose titration was not included as a cost input for apomorphine sublingual film and apomorphine hydrochloride injection treatment and this could also increase total costs.

A further limitation of this study relates to the fact that efficacy parity could only be assumed for the comparisons of apomorphine sublingual film and apomorphine hydrochloride injection in the model, owing to a lack of direct head-to-head comparisons and differences in study design, which made an indirect, anchor-based comparison difficult to perform. As both “on-demand” treatments are different formulations of the same molecule (ie, apomorphine), both formulations were expected to have similar efficacy. More head-to-head studies are needed to further inform the comparative effectiveness and cost-effectiveness of “on-demand” treatments for “OFF” episodes in patients with PD.

With regard to treatment discontinuation inputs, the apomorphine hydrochloride injection 3-month discontinuation rate was not available in the literature and was, therefore, computed using a formula based on the discontinuation rate observed in the apomorphine sublingual film clinical study data. Because the approach fit the apomorphine sublingual film study data well, it was assumed reasonable for use to project the discontinuation rate for apomorphine hydrochloride injection. However, it should be noted that this is only an assumption and may constitute a limitation of this analysis.

Scenario analyses addressed some of the limitations related to model inputs. Scenario 1 addressed the limited efficacy data available by using results from a second indirect treatment comparison of apomorphine sublingual film and levodopa inhalation powder. Scenario 2 relaxed an assumption regarding the functional form of utility. Scenario 3 addressed uncertainty with regard to the relevant time horizon, given the advanced age of patients and that many patients will discontinue treatment or die within 5 years. Finally, scenario 4 included cost differences related to informal caregiving. Informal caregiving included care provided by nonprofessional caregivers (ie, family and friends) and not institutional caregivers (ie, long-term care facilities, including skilled nursing facilities). While informal caregiver costs were not borne by payers, they reflected a real burden to families. Differences in “OFF” times across treatments were related to caregiver burden in this scenario.

## CONCLUSIONS

The results of this cost-effectiveness analysis model suggest that, from a US payer perspective, apomorphine sublingual film represents a cost-effective option compared with apomorphine hydrochloride injection and levodopa inhalation powder for the treatment of “OFF” episodes in patients with PD.

### Disclosure of Conflicts of Interest

AT, EP, and GRW are employees of Sunovion Pharmaceuticals Inc. (Marlborough, MA, USA) and hold stock/options. NK, MLZ, and ID are employees of Analysis Group, Inc. (Boston, MA, USA), which has received consulting fees from Sunovion Pharmaceuticals Inc.

### Data Sharing Statement

Data for this study will be made available upon request via the Clinical Study Data Request site (http://clinicalstudydatarequest.com).

## Supplementary Material

Online Supplementary Material This supplementary material has been provided by the authors to give readers additional information about their work.
