# Supplementary material for: Cost-Effectiveness of Apomorphine Sublingual Film as an “On-Demand” Treatment for “OFF” Episodes in Patients with Parkinson’s Disease
Source: J Health Econ Outcomes Res. 2021 Nov 17;8(2):82–92. doi: 10.36469/jheor.2021.29488 (PMC8809356; doi:10.36469/jheor.2021.29488)
Supplement: Online Supplementary Material — This supplementary material has been provided by the authors to give readers additional information about their work. [file jheor_2021_8_2_29488_75060.pdf]

Journal of Health Economics and Outcomes Research

Online Supplementary Material

Thach A, Kirson N, Zichlin ML, Dieye I, Pappert E, Williams GR. Cost-Effectiveness of Apomorphine Sublingual Film as an "On-Demand" Treatment for "OFF" Episodes in Patients with Parkinson's Disease. *JHEOR*. 2021;8(2):110-120. doi:10.36469/jheor.2021.29488

Table S1. Mortality Hazard Ratios Based on Age and Disease Stage Incorporated in the Model
Table S2. Treatment-Emergent AEs Reported by ≥5% of Patients in Any Comparator, and Related Health-care Use, Unit Cost, and Utility Decrements Incorporated in the Model
Table S3. Medical Costs Incorporated in the Model
REFERENCES

This supplementary material has been provided by the authors to give readers additional information about their work.

This is an open-access article distributed under the terms of the Creative Commons Attribution 4.0 International License (CCBY-4.0). View this license's legal deed at http://creativecommons.org/licenses/by/4.0 and legal code at http://creativecommons.org/licenses/by/4.0/legalcode for more information.

Table S1. Mortality Hazard Ratios Based on Age and Disease Stage Incorporated in the Model

Age	Hoehn and Yahr Stage	Hazard Ratio	95% CI
<65 years	1	1.00	–
	2	3.09	1.66, 5.76
	3	2.47	1.17, 5.21
	4	13.83	1.94, 98.84
	5	13.83	1.94, 98.85
≥65 years	1	1.00	–
	2	2.03	1.41, 2.94
	3	2.16	1.43, 3.26
	4	4.99	3.09, 8.05
	5	4.99	3.09, 8.06

Probability of death was calculated from the United States life tables[1] with an adjustment based on the relative risk of death given the patients' age and Parkinson's disease severity, informed by Liou et al.[2]

Abbreviations: CI, confidence interval.

Table S2. Treatment-Emergent AEs Reported by ≥5% of Patients in Any Comparator, and Related Health-care Use, Unit Cost, and Utility Decrements Incorporated in the Model

AE	Apomorphine Sublingual Film (%)	Apomorphine Hydrochloride Injection (%)	Levodopa Inhalation Powder (%)	Unit Cost (USD)	Utility Decrement
Nausea/Vomiting	27.78	30.00	5.26	$999	–
Oral mucosal erythema	7.41	–	–	$87	–
Somnolence	12.96	35.00	–	$87	–
Dizziness	9.26	20.00	0.88	$331	0.16
Headache	5.56	–	–	$0	–
Yawning	3.70	40.00	–	$0	–
Rhinorrhea	7.41	20.00	–	$87	–
Fatigue	7.41	–	–	$87	–
Fall	5.56	–	2.63	$90	–
Confusion/Hallucination	1.85	10.00	–	$236	–
Hyperhidrosis	5.56	–	–	$87	–
Dry mouth	5.56	–	–	$0	–
Cough	1.85	–	14.91	$345	–
Laceration	5.56	–	–	$0	–
Edema[a]	11.11	10.00	–	$90	–
Injection site reactions	0	45.00	–	$166	–
Upper respiratory tract infection	–	–	6.14	$87	–
Chest pain/pressure/angina	0	0.15	–	$87	0.18

All costs are presented in 2020 USD. AE incidence rates were extracted from CTH-300 study data for apomorphine sublingual film,[3] from a CADTH Pharmacoeconomic Report for apomorphine hydrochloride injection,[4] and from LeWitt et al for levodopa inhalation powder.[5] AE costs were derived from the CADTH Pharmacoeconomic Report[4] or assumed to be similar, if missing.

Abbreviations: AE, adverse event; CADTH, Canadian Agency for Drugs and Technologies in Health; USD, United States dollars.
[a] Edema in the apomorphine hydrochloride injection pivotal study was related to the extremities, while for the CTH-300 study, it was related to oral events.[3,6]

Table S3. Medical Costs Incorporated in the Model

	Baseline Units	Units Associated With One Extra "OFF" Hour	Cost per Unit (USD)
Hospitalization, days	0.016	0.004	$960
Specialist visits, n	0.653	0	$166
ER visits, n	0.028	0.006	$973
Informal caregiver, hours[a]	444.222	18.759	$43

All costs are presented in 2020 USD. Baseline HRU and HRU associated with one extra "OFF" hour were calculated based on data from Adelphi Real World's Disease Specific Programmes for PD; costs per unit were derived from Palmer et al.[7] Informal caregiver hours were based on Findley et al,[8] with the cost per unit derived from the US Bureau of Labor Statistics.[9]

Abbreviations: ER, emergency room; HRU, health-care resource utilization; US, United States; USD, United States dollars.
[a] Included in a scenario analysis.

REFERENCES

1. United States Centers for Disease Control and Prevention. United States Life Tables. https://www.cdc.gov/nchs/products/life_tables.htm. Accessed March 3, 2021.
2. Liou HH, Wu CY, Chiu YH, et al. Mortality of Parkinson's disease by Hoehn-Yahr stage from community-based and clinic series [Keelung Community-based Integrated Screening (KCIS) no. 17)]. *J Eval Clin Pract*. 2009;15(4):587-591. doi:10.1111/j.1365-2753.2008.01041.x
3. Olanow CW, Factor SA, Espay AJ, et al.; for the CTH-300 Study investigators. Apomorphine sublingual film for off episodes in Parkinson's disease: a randomised, double-blind, placebo-controlled phase 3 study. *Lancet Neurol*. 2020;19(2):135-144. doi:10.1016/S1474-4422(19)30396-5
4. Clinical Review Report: Apomorphine (Movapo): (Paladin Labs Inc.). Indication: The acute, intermittent treatment of hypomobility "off" episodes ("end-of-dose wearing off" and unpredictable "on/off" episodes) in patients with advanced Parkinson's disease. Ottawa (ON): Canadian Agency for Drugs and Technologies in Health. In: CADTH Common Drug Review. https://www.ncbi.nlm.nih.gov/books/NBK534020/. Published 2018. Accessed March 2, 2021.
5. LeWitt PA, Hauser RA, Grosset DG, et al. A randomized trial of inhaled levodopa (CVT-301) for motor fluctuations in Parkinson's disease. *Mov Disord*. 2016;31(9):1356-1365. doi:10.1002/mds.26611
6. Dewey RB Jr, Hutton JT, LeWitt PA, et al. A randomized, double-blind, placebo-controlled trial of subcutaneously injected apomorphine for parkinsonian off-state events. *Arch Neurol*. 2001;58(9):1385-1392. doi:10.1001/archneur.58.9.1385
7. Palmer CS, Nuijten MJ, Schmier JK, Subedi P, Snyder EH. Cost effectiveness of treatment of Parkinson's disease with entacapone in the United States. *Pharmacoeconomics*. 2002;20(9):617-628. doi:10.2165/00019053-200220090-00005
8. Findley LJ, Lees A, Apajasalo M, Pitkänen A, Turunen H. Cost-effectiveness of levodopa/carbidopa/entacapone (Stalevo) compared to standard care in UK Parkinson's disease patients with wearing-off. *Curr Med Res Opin*. 2005;21(7):1005-1014. doi:10.1185/030079905X49653
9. United States Bureau of Labor Statistics. Employer costs for employee compensation. National Compensation Survey, March 2004-September 2018. https://www.bls.gov/ect/. Published 2019. Accessed March 2, 2021.
